# Polyphenol-Rich Lentils and Their Health Promoting Effects

**DOI:** 10.3390/ijms18112390

**Published:** 2017-11-10

**Authors:** Kumar Ganesan, Baojun Xu

**Affiliations:** Food Science and Technology Program, Beijing Normal University-Hong Kong Baptist University United International College, Zhuhai 519085, China; kumarganesan@uic.edu.hk

**Keywords:** polyphenols, lentils, antioxidants, degenerative diseases, health-promoting effects

## Abstract

Lentil (Lens culinaris; Family: Fabaceae) is a potential functional dietary ingredient which has polyphenol-rich content. Several studies have demonstrated that the consumption of lentil is immensely connected to the reduction in the incidence of diseases such as diabetes, obesity, cancers and cardiovascular diseases due to its bioactive compounds. There has been increasing scientific interest in the study area of lentils as the functional food due to its high nutritive value, polyphenols, and other bioactive compounds. These polyphenols and the bioactive compounds found in lentil play an important role in the prevention of those degenerative diseases in humans. Besides that, it has health-promoting effects. Based on the in vitro, in-vivo and clinical studies, the present review focuses to provide more information on the nutritional compositions, bioactive compounds including polyphenols and health-promoting effects of lentils. Health-promoting information was gathered and orchestrated at a suitable place in the review.

## 1. Introduction

Lentil (*Lens culinaris*; Family: Fabaceae) is an annual indigenous plant from Western Asia and other parts of the world, including North America. Furthermore, this species is now diversified from Hindukush to Afghanistan and Ethiopia to Mediterranean countries [1]. It is well known for its lens-shaped edible seed, which has the most significant dietary compositions, containing macro- and micro-nutrients [2]. Lentils exist as a spectrum of colors, which includes yellow, orange, red, green, brown or black, depending on the cultivar, the composition of the seed coats and cotyledons [3]. The color of dehulled seeds is mainly associated with the cotyledon color, which could be yellow, red or green. While the color of the intact seed is based on the seed coat, it could be tan, brown, green, gray or black. The seed coats of lentil have a higher amount of flavan-3-ols, proanthocyanidins and some flavonols. This suggests that lentil featuring green and gray seed coats might be more promising for a health-promoting diet. According to the Food and Agriculture Organization statistics report in 2014, the global production of the lentils was primarily cultivated and harvested by Canada and India, which were estimated to be 1.99 million and 1.1 million metric tons, followed by Turkey (0.34 million), Nepal (0.22 million) and China (0.125 million) [4]. The evidence demonstrated that the consumption of lentils is highly associated with reductions in the incidence of degenerative diseases including diabetes, cardiovascular disease (CVD) and cancers. There has been an increase in scientific interest of the study of lentils as a functional food due to their high nutritional compositions, nutritive value and the presence of bioactive secondary metabolites. These bioactive compounds in lentils play a vital role in the prevention of degenerative diseases in humans and a significant role in improving health. Based on the explorative studies, the current comprehensive review aims to provide information on the nutritive compositions, bioactive compounds and health-promoting effects of polyphenol-rich lentils and explores their therapeutic values for future clinical studies. 

## 2. Materials and Methods

An electronic search was conducted using PubMed, Science Direct and Google Scholar by finding the keywords “Lentils” AND “bioactive compounds” AND “nutritional compositions” AND “polyphenols” OR “antidiabetic” OR “antioxidants” OR “antimicrobial” in “Title/Abstract/Keywords”, without date restriction, to identify all published studies (in vitro, in vivo, clinical and case-control) that have investigated the connection between lentils and their various beneficial effects. Health-promoting information was gathered and orchestrated in the suitable place in the review.

## 3. Nutritional Compositions of Edible Lentils

Nutritional compositions of raw, sprouted and cooked lentils are summarized in Table 1. Lentils are known to be an abundant source of protein storage, providing essential and non-essential amino acids to the human body. The predominant proteins in lentils are globulin (47% of the total seed proteins) and an adequate quantity of albumin [5]. Lentils play an important role in crop rotation and the ability to fix atmospheric nitrogen. High quantities of these proteins and essential amino acids in lentils offer an important dietary source for low and middle-income countries [6]. Among 23 pulses, lentils yield the second highest starch percentage of 47.1% and a greater percentage of insoluble dietary fibers [7,8]. Lentils are known to be a good source of prebiotics [9] and have nutritionally important quantities of prebiotic carbohydrates (12.3–14.1 g/100 g of dry lentils) that help to keep up the gut microbial environment and prevent gut-associated diseases [10,11]. Furthermore, lentils are relatively low in fat and sodium, but high in potassium content (1:30 ratio of sodium and potassium) [12]. Given that, it is the best dietary food for patients with obesity and CVD. Lentil seeds are an excellent vegetable source of iron. Studies have shown that the consumption of cooked lentil in the diet prevents iron deficiency anemia [13], iron being a very important mineral, which is required daily, especially for adolescents and pregnant women. Several minerals (zinc, copper, manganese, molybdenum, selenium and boron) and vitamins (thiamine, riboflavin, niacin, pantothenic acid, pyridoxine, folate, α, β and γ tocopherols and phylloquinone) have been well documented in lentils [7,14,15]. Furthermore, lentils have an average quantity of vitamin K of 5 μg/100 g, as reported by the United States Department of Agriculture (USDA) [7]. However, the daily requirement of this vitamin in adults is about 80 μg. The low content of vitamin K renders lentils as safe for patients with CVD upon anticoagulant treatment. Overall, lentils are considered as one of the best dietary sources that has health-promoting effects on various illnesses.

## 4. Bioactive Compounds in Lentils

Various bioactive compounds or secondary metabolites are present in the lentil seed, which are categorized into different functional groups. The bioactive functional groups and their quantity in lentils are listed in Table 2.

## 5. Polyphenols in Lentils

Lentils have the highest total phenolic content in comparison to six other common legumes, such as green pea, chickpea, cowpea, yellow pea, mung bean and peanut [3]. Polyphenols are generally a large group of compounds, classified into different classes, based on the presence of the number of phenolic rings and their structural elements or substituents [30,31]. Two main groups can be identified based on the aromatic rings, which are attached to the heterocyclic rings, known as the flavonoid groups (flavones, flavonols, flavanones, flavanonols, flavanols or catechins, anthocyanins, neoflavonoids and chalcones) and the non-flavonoid groups (simple phenols, phenolic acids, hydroxybenzoic acids, tannins, acetophenones and phenylacetic acids; hydroxycinnamic acids, coumarins, benzophenones, xanthones, stilbenes, lignans and secoiridoids) [31,32]. Various functional polyphenols in the lentils are described according to their classes, subclasses and chemical structures in Table 3.

## 6. Health Promoting Effects of Lentils

Polyphenol-rich lentils have potential health benefits as complementary and alternative medicines, which are exerted in the form of antioxidant, antibacterial, anti-fungal, antiviral, cardioprotective, anti-inflammatory, nephroprotective, antidiabetic, anticancer, anti-obesity, hypolipidemic and chemopreventive activities. Furthermore, lentils are useful as a prognostic marker for various cancers including thyroid and hepatic carcinoma. Detailed information on lentil polyphenols’ dose range, route of administration, model used and negative controls is presented based on in vivo, in vitro and clinical research studies according to the title and is depicted in Table 4, Table 5, Table 6, Table 7 and Table 8.

### 6.1. Anti-Diabetic Activity of Lentils

Świeca et al. [38] observed that the regular consumption of the germinated lentils is beneficial for the prevention and management of diabetes. Lentils have the ability to improve blood glucose, lipid and lipoprotein metabolism in diabetic and healthy human beings [39]. In vitro and in vivo studies of polyphenol-rich lentil seed showing the anti-diabetic potentials are summarized in Table 4. Besides that, the studies that are associated with lentils and diabetic animal models have reported that the high flavonoid and fiber content of lentils play a significant role in the gut motility and prevent the impairment of metabolic control in diabetic rats, so having a promising implication for diabetic patients [40]. The regular consumption of cooked lentils (50 g) among diabetic patients leads to significant reductions of fasting blood sugar (FBS), glycemic load and glycemic index in streptozotocin (STZ)-induced diabetic animals [41,42]. Reductions of the glycemic index from the diet are due to the presence of polyphenols in the lentils that have been linked with health-promoting impacts on metabolic disorders such as diabetes, obesity, coronary heart diseases and CVD [43,44]. Furthermore, in vitro and in vivo studies have also demonstrated that lentils in the diet regulate starch digestibility, glycemic load and the glycemic index, which diminish diabetes complications [45,46]. Thus, a diet including lentils appears to be an effective intervention and management strategy for the prevention of diabetes.

### 6.2. Antioxidant Potential of Lentils

A wide range of in vitro evidence implies that lentils have the highest total antioxidant capacity when they are compared to chickpeas, common beans and soybeans, which were measured by 2,2-diphenyl-1-picrylhydrazyl (DPPH), ferric reducing antioxidant power, oxygen radical absorbing capacity, Trolox equivalent antioxidant capacity and total radical-trapping antioxidant parameters [51,52,53,54]. Evidence has shown that lentils have greater oxygen radical scavenging potential compared to various vegetables and fruits, such as onion, horseradish, potatoes, wheat germ, blueberries and sweet cherries [7]. Lentils have different groups of phenolic compounds such as procyanidin and prodelphinidin dimers and trimers, gallate procyanidins, kaempferol derivatives, quercetin glucoside acetate, luteolin derivatives and *p*-coumaric acid, hydroxybenzoic compounds, protocatechuic, vanillic acid, aldehyde *p*-hydroxybenzoic, trans-ferulic acid and trans-*p*-coumaric acid, compared to other legumes, providing greater antioxidant potentials and health-promoting effects. These phenolic compounds in lentils naturally act as antioxidants and have the ability to restrict the formation of reactive oxygen species, as well as superoxide anion by chelating metal ions or inhibiting enzymes [52,53]. In vitro and in vivo studies of polyphenol-rich lentils that exert antioxidant potentials are summarized in Table 5.

### 6.3. Anti-Obesity Activity of Lentils

Large prospective epidemiological studies have reported that the intake of phenolic-rich lentils is inversely connected with the incidence of obesity and diabetes [55]. An earlier human study shows that the intake of lentil seed along with pasta and sauce reduces food intake, body weight and waist circumference [56]. Furthermore, lentil seed containing flavonoids and fiber enhances satiety and lowers the amount of food intake, which lead to maintaining body weight in obese subjects [56]. Observational studies have further reported an inverse relationship between the consumption of lentils and the basal metabolic index or risk associated with obesity [57]. Besides that, interventional studies have shown the potential of lentils to inhibit α-glucosidase and pancreatic lipase, which has the ability to decrease glucose and fat digestion and absorption in the intestine. Ultimately, polyphenol-rich lentils control postprandial glucose and fat, which is crucial in the management of diabetes and obesity [58,59]. Flavonoids in lentils have the potential to inhibit the actions of α-glucosidase and lipase, which suggests that dietary lentil consumption could manage post-prandial blood glucose and body weight [37]. In vitro, in vivo, clinical and interventional/observational studies of lentils possessing anti-obesity potentials are summarized in Table 6.

### 6.4. Cardioprotective Effect of Lentils

Phenolic-rich lentil seed consumption has been inversely linked with the occurrence of various CVDs [43]. Lentils containing polyphenols have the potential to reduce blood pressure by angiotensin I-converting enzyme (ACE) inhibitor activity [86,87]. The recent study observed that bioactive compounds (legumin, vicilin and convicilin) in lentil possess higher antioxidant, ACE-inhibitory and cardioprotective activity [88]. Besides that, the polyphenol-rich lentil seeds have the ability of antihyperlipidemic, hypohomocysteinemic, anti-cholesterolemic and a cardioprotective effect that reduces the risk of hypertension and coronary artery diseases [76,82]. In the hypertensive animal model, administration of lentils actively reduces the total cholesterol (TC), triglycerides (TG), low density lipoprotein (LDL) and pathological manifestations of cardio-morphometric analysis. These findings reinforce the importance of lentil seed and its diet prescription as a therapeutic potential for hypertensive patients [78,84]. Al-Tibi et al. [42] observed that treatment with lentil seeds reduces the glycemic index and hyperlipidemic effects in the STZ-induced diabetic animal model. In this study, lentils significantly raised the high density lipoprotein (HDL) levels and reduced blood glucose levels in diabetic rats. Concisely, these studies recommend that the dietary consumption of polyphenol-rich lentils should be on a regular basis, having the potential to decrease the risk of cardiovascular and coronary artery diseases. In vitro and in vivo studies of lentils exerting cardioprotective potentials are summarized in Table 6.

### 6.5. Antimicrobial Activity of Lentils

Lentils containing flavonoids and lectins have been reported as non-toxic and safe for use in medical diagnostic kits [89]. A bioactive peptide called “defensing”, which is isolated from germinated lentil seeds, possesses a broad spectrum of biological activities, including antimicrobial activities against various infections associated with bacteria and fungi [21,90]. It is a group of “host defense peptides” synthesized in the lentil seeds, which are involved in the development of innate immunity. They are tiny, basic, cysteine-rich peptides, containing antifungal activity, which inhibit the growth of *Aspergillus niger* [21,91]. Likely, “defensins” can interrupt viral digestive enzymes, such as human immunovirus (HIV)-1 reverse transcriptase, which impacts viral replication. “Defensins” have been further observed to block ion channels and to inhibit protein translation. Therefore, “defensing” in lentil seeds along with phenolic compounds acts as a potential inhibitor of microbial growth. In vitro studies of lentils exerting antimicrobial potentials are summarized in Table 7.

### 6.6. Anticancer Activity of Lentils

The consumption of lentil seeds reduces the incidence of various cancers including colon, thyroid, liver, breast and prostate [97,98,99]. A large prospective epidemiologic study associated with polyphenol-rich lentils and breast cancer on 90,630 women exhibited an inverse relationship between lentils and the risk of breast cancer [98]. Lentil seeds have a high polyphenolic content that potentially could prevent carcinogens through chemo-preventive activities, including the uptake of carcinogens, activation or formation, detoxification, binding to DNA and fidelity of DNA repair [100,101]. Moreover, lectins in lentils have anticancer properties, which have been observed in various in vitro, in vivo and human studies [20]. These lectins along with phenolic compounds in lentil seeds have been proven as therapeutic agents. They potentially bind to cancer cell membranes/receptors, causing cytotoxicity, apoptosis and autophagy; thereby, they inhibit the growth of tumors [20]. The underlying mechanism of the anticancer potential of lectins and phenolic compounds in lentil is that they bind to ribosomes, which inhibits protein synthesis. Furthermore, this provokes a change of the cell cycle by inducing non-apoptotic G1-phase accumulation mechanisms, G2/M phase cell cycle arrest and apoptosis. In addition to that, this can also activate the caspase cascade in mitochondria and downregulate telomerase activity, which inhibits angiogenesis [20,102]. Thus, lectins and phenolic compounds derived from lentil seeds seem to be promising therapeutic agents against tumorigenesis or cancer cell agglutination and/or aggregation. The lentil seeds and their chemo-preventive potential on colorectal carcinogenesis have been well documented using azoxymethane, significantly reducing the number of dysplastic lesions and neoplasms in the colon of rats [101,103]. In addition, lentils have greater chemopreventive potential when compared to green and yellow peas [104]. This is because lentils contain antioxidant bioactive compounds such as flavonoids (flavanones, flavan-3-ols, flavones, flavonols, anthocyanidins and tannins, including condensed tannins or proanthocyanidins) that are greatly responsible for chemoprevention. This chemo-preventive potential is not constrained to polyphenolic-rich lentils or split seeds. In vitro and in vivo studies of lentil seeds exerting anticancer and chemopreventive potentials are summarized in Table 8.

## 7. Conclusions

Lentils have been consumed as a part of the diet worldwide and play a significant function in human nutrition as a rich source of bioactive and non-bioactive nutrients. When comparing to pulses, lentils have the highest starch content and insoluble dietary fiber content and high quantities of prebiotic carbohydrates that maintain the gut microbiota, which prevents colon-associated diseases. Lentils are among the cost-effective legumes, and they have lower quantities of fat, sodium and vitamin K, but a high content of potassium. This demonstrates them as a health-promoting source of nutrients, and their intake in the daily diet should increase, as this is related to the prevention of obesity and CVD. Besides these nutrients, lentils have certain bioactive food components, namely “polyphenols”. These polyphenol-rich lentil seeds have antioxidant potential and a primary function in protecting against various diseases such as diabetes, obesity, CVD and cancer. Various rodent studies and large prospective epidemiologic studies have reported that lentil consumption reduces the risk of those chronic diseases, which could be an exceptionally cost-effective approach towards improving health. Due to their nutritional and health-promoting potential, the development of lentil-based functional food products as well as nutraceuticals should be widely promoted.

## Figures and Tables

**Table 1:** Nutritional compositions of lentils in 100 g of the edible portion [7].

Nutrients	Unit	Raw	Sprouted	Cooked
Water	g	8.26–9.65	51.85–67.34	69.64–137.89
Energy	kcal	343–356	82–106	116–226
Protein	g	24.44–25.71	6.9–8.96	9.02–17.86
Total lipid (fat)	g	0.92–1.06	0.42–0.55	0.38–0.75
Carbohydrate	g	60–64.44	17.05–22.14	20.13–38.69
Total dietary fiber	g	10.7–31.4	-	7.9–15.6
Total sugars	g	2.03–2.86	-	1.80–3.56
**Minerals**				
Calcium	mg	35–57	19–25	19–38
Iron	mg	6.51–7.71	2.47–3.21	3.33–6.59
Magnesium	mg	47–69	28–37	36–71
Phosphorus	mg	281–335	133–173	180–356
Potassium	mg	677–943	248–322	369–731
Sodium	mg	3–6	8–11	123–471
Zinc	mg	3.27–5.89	1.16–1.51	1.27–2.51
**Vitamins**				
Vitamin C	mg	3.4–4.5	12.7–16.5	1.5–3.0
Thiamin	mg	0.756–0.873	0.176–0.228	0.169–0.335
Riboflavin	mg	0.189–0.211	0.099–0.128	0.073–0.0145
Niacin	mg	2.605–3.459	0.869–1.128	1.060–2.099
Vitamin B6	mg	0.540–0.698	0.146–0.190	0.178–0.352
Folate	µg	479–555	77–100	181–358
Vitamin B12	µg	0.00	0.00	0.00
Vitamin A, RAE	µg	2.0–2.5	1.8–2.0	0
Vitamin A, IU	IU	32–39	35–45	8–16
Vitamin E	mg	0.49–0.55	0	0.11–0.22
Vitamin K	µg	4.2–5.0	0	1.7–3.4
**Lipids**				
Total saturated fatty acids	g	0.154–0.198	0.044–0.057	0.053–0.105
Total monounsaturated fatty acids	g	0.0179–0.193	0.08–0.104	0.064–0.127
Total polyunsaturated fatty acids	g	0.469–0.526	0.169–0.219	0.175–0.346

**Table 2:** List of bioactive functional groups in lentils and their biological functions.

Bioactive Functional Groups	Individual Components	Quantity in 100 g of Lentils	Biological Functions	Reference
Phytosterols	β-sitosterol	15.0–24.0 mg	Regulate the membrane fluid	[14,16]
	campesterol	15.0 mg		
	stigmasterol	20.0 mg		
**Active Proteins**				
Trypsin/protease inhibitors	Bowman–Birk trypsin inhibitors	3–8 trypsin inhibitor unit (TIU)/mg	Anti-nutritional components; decrease the digestibility of dietary proteins; inhibit the cell proliferation in cancer	[17,18]
Lectins	Lectins or hemagglutinins	12.0 mg	Ability to agglutinate red blood cells RBC and strong stimulators of murine B lymphocyte proliferation	[19,20]
Defensins	Defensins	8.0 mg	Participate in the development of innate immunity	[21]
Dietary Fibers	Fibers	Insoluble fibers (93–99.7 mg/g) and soluble fibers (<7 mg/g)	Potential effect of hypocholesterolemic, anti-cancer, anti-tumor, antibacterial and hypoglycemic effects	[7,22]
	Resistant starches	25.4 g	Significant contributor to gastrointestinal health and gut microbiota	[23]
Polyphenols Flavonoids	Flavonols (e.g., quercetin and kaempferol)	0.03 to 10.85 and 0.24 to 13.20 mg	Antioxidant potential	[3,24]
	Flavones, flavanones	Total phenolic content: 26 mg gallic acid equivalents (GAE/100 g fresh wt; total flavonoid content: 21 mg catechin equivalents/100 g, and the condensed tannin content of 870 mg catechin equivalents/100 g	Antioxidant activity and potential effect on cardiovascular disease (CVD), diabetes, osteoporosis and neurodegenerative diseases	[24,25]
	Proanthocyanidins or condensed tannins (e.g., prodelphinidins and procyanidins)			
	Flavan-3-ols or flavanols (e.g., catechin and gallocatechin)	759 mg (GAE)/100 g; glycosides of flavanones: 33.1–186.0 µg; glycosides of flavonols: 9.6–241 µg; dimers procyanidins: 619–1122 µg; trimer procyanidins: 441–498 µg; tetramer procyanidins: 18.5–59.5 µg; galloylated procyanidins 69.3–123 µg	Antioxidant activity	[3,24]
	Anthocyanidins (e.g., delphinidin and cyanidin)			
Polyphenols Non-flavonoids	Hydroxybenzoic acids	Hydroxybenzoic acids: 4.5–28.4 µg	Antioxidant activity and potential effect on diabetes, osteoporosis CVD and neurodegenerative diseases	[24,25]
	Hydroxycinnamic acids (e.g., *p*-coumaric acid, ferulic acid and sinapic acid)	Prodelphinidins 369–725 µg; condensed tannins: 870 mg catechins equivalent	Antioxidant activity	[3,24]
	Stilbenoids, trans-resveratrol-3-*O*-glucoside	Glycosides of trans-resveratrol: 5.5–9.3 µg;	Antioxidant activity and potential effect on diabetes and CVD	[24,25]
Phytoestrogens: isoflavones	Formononetin, daidzein, genistein, glycitein, matairesinol, biochanin A, coumestrol, lariciresinol, pinoresinol, secoisolariciresinol, coumestrol	Total isoflavones (9.5 μg), total lignans (26.6 μg) and total phytoestrogens (36.5 μg)	Antioxidant potential	[26]
Phytate	Phytic acid	620 mg	Inhibit the proliferation of colorectal cancer	[27]
Triterpenoids	Squalene	0.7 mg	Chemopreventive potential against colorectal cancer	[28]
Saponins	Saponins	25 mg	Hypoglycemic and antidiabetic potential	[29]

**Table 3:** List of polyphenols in lentils (*Lens culinaris*) [30,31,32,33,34,35,36,37].

Polyphenol	Classes	Sub-Classes	Compound Name	Structure
Flavonoids	Flavonoids	Flavanols	(−)-Epigallocatechin	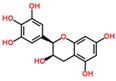
			(+)-Catechin-3-*O*-glucose	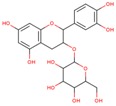
			Catechin	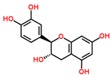
			Catechin-7-*O*-glucoside	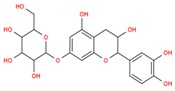
			Catechin gallate	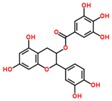
			Epicatechin	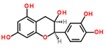
			Epicatechin gallate	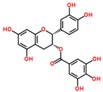
		Flavonols	Quercetin-3-*O*-glucoside	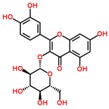
			Quercetin-3-*O*-galactoside	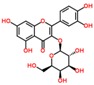
			Quercetin-3-*O*-xyloside	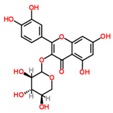
			Kaempferol-3-*O*-rutinoside 7-*O*-rhamnoside	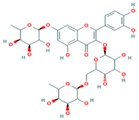
			Kaempferol-4′-*O*-glucoside	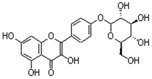
			Kaempferol-5-*O*-glucoside	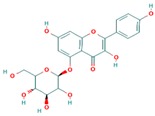
			Kaempferol-3-*O*-glucoside	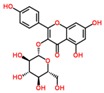
			Kaempferol-3-*O*-rutinoside	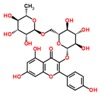
			Myricetin-3-*O*-rhamnoside	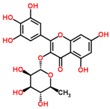
			4″″-Acetylsagittatin A	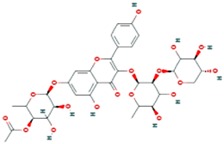
		Proanthocyanidins	Procyanidin	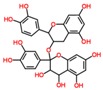
			Prodelphinidin	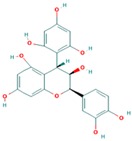
		Flavanones	Eriodictyol	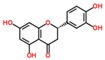
			Eriodictyol-7-*O*-rutinoside	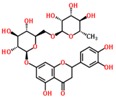
			Naringenin	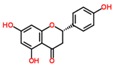
		Flavone	Luteolin	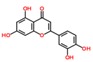
			Luteolin-4′-*O*-glucoside	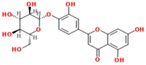
			Luteolin-3′,7-diglucoside	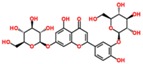
			Luteolin-7-*O*-glucoside	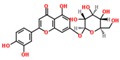
			5,7-dimethoxyflavone	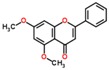
		Anthocyanins	Malvidin-3-*O*-galactoside	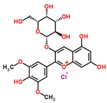
Non-flavonoids	Phenolic acids	Hydroxybenzoic acids	Syringic acid	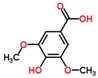
			Vanillic acid 4-|A-D-glucoside	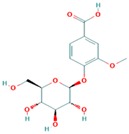
			2,3-Dihydroxy benzoic acid	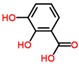
			*p*-hydroxy benzoic acid	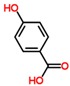
			Gallic acid	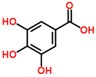
		Hydroxycinnamic acid	3-hydroxy cinnamic acid	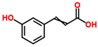
			*p*-Coumaroyl malic acid	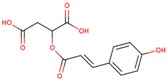
			Sinapic acid	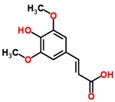
	Other polyphenols	Hydroxycoumarin	4-Hydroxy-6-methyl coumarin	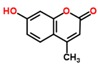

**Table 4:** Summary of in vitro, in vivo and clinical studies on the antidiabetic activities of polyphenol-rich lentils.

Polyphenol-Rich Lentils	Model	Dose and Route of Administration	Negative Control	Investigation	Results	Reference
Total phenolics and flavonoids	In vitro	50–500 μg/mL	-	Assay of antioxidant activities DPPH, FRAP, ORAC and inhibitory properties against α-glucosidase and pancreatic lipase	Antidiabetic, hypotensive and antioxidant activity	[47]
Total phenolics	In vitro	100.9 mg/g f.m.	300 mM NaCl	Assay of α-amylase inhibitor activity and expected glycemic index values	Antidiabetic potential	[38]
Flavonoids	Male albino rats	15 g/kg/p.o. of lentil food formulation	Alloxan (150 mg/kg bw	Assay of glucose, urea, serum total protein, total TG and TC	Antidiabetic and hypolipidemic potential	[48]
Total phenolics and flavonoids	Male Nile rats	720 g/kg/p.o. of lentil food formulation	STZ (35 mg/kg i.p.)	Assay of glycemic index, glycemic load and cumulative load, blood glucose (fasting, random and OGTT) and plasma lipid parameters (plasma TC and TG) plus necropsy findings (liver and kidney pathology plus adipose reserves)	Antidiabetic and hypolipidemic potential	[49]
Flavonol glycosides and free flavanols	Male Sprague-Dawley rats	57% raw whole lentil; 52% cooked whole lentil; 51% raw dehulled lentil; 47% cooked dehulled lentil/p.o.	STZ (35 mg/kg i.p.)	Assay of serum glucose and serum lipid levels	Antidiabetic and hypolipidemic potential	[41,42]
Total phenols	Human with diabetes	50 g cooked lentil/p.o.	-	Assay of FBS, TC and glycemic control	Antidiabetic and cardioprotective activity	[41]
Total phenols	Human with diabetes	1 cup cooked lentil/day/p.o.	-	Assay of body weight, HbA1C, TC, BP, heart rate, glycemic control	Antidiabetic and cardioprotective activity	[50]
Total phenolics and flavonoids	Obese patients with type 2 diabetes	60 g lentil sprouts/p.o. daily during 8 weeks	-	Assay of weight, height and waist circumference, lipid profile,	Antidiabetic and hypolipidemic potential	[39]

DPPH: 2,2-diphenyl-1-picrylhydrazyl; FRAP: ferric reducing antioxidant power assay; ORAC: oxygen radical absorbance capacity; g f.m.: germination fraction matter; p.o.: per oral; i.p.: intraperitoneal; bw: body weight; STZ: streptozotocin; OGTT: oral glucose tolerance test; TG: triglycerides; TC: total cholesterol; FBS: fasting blood sugar; BP: blood pressure; HbA1C: glycated hemoglobin.

**Table 5:** Summary of in vitro, in vivo and clinical studies on the antioxidant activities of polyphenol-rich lentils.

Polyphenol-Rich Lentils	Model	Dose and Route of Administration	Negative Control	Investigation	Results	Reference
Procyanidin and prodelphinidin dimers and trimers; gallate procyanidins; kaempferol derivatives, quercetin glucoside acetate; luteolin derivatives and *p*-coumaric acid	Human astrocytoma cell line (U-373), renal adenocarcinoma (TK-10), breast adenocarcinoma (MCF-7), melanoma (UACC-62), colon carcinoma (HT29) and hepatocellular carcinoma (HepG2)	0.06–0.12 µg/µL	H_2_O_2_, FeSO_4_ and FeSO_4_ + H_2_O_2_	Assay of antioxidant activity by ORAC, DPPH, MTT and intracellular ROS	Antioxidant neuroprotective and anticancer activities	[60]
Flavanols and phenolic acids	Human colonic carcinoma cell line (Caco-2)	20–100 μg/mL	-	Assay of proinflammatory cytokines COX-2, IL-1β and IL-6 in TNF-α	Anti-inflammatory activity	[61]
Total phenolics and flavonoids	In vitro	200 mg sprout extracts	-	Assay of radical activity and expected glycemic index values	Antioxidant and antidiabetic activity	[62]
Flavonoids	In vitro	100 μL, 1 mg/mL	-	Assay of TEAC, DPPH, superoxide radical, hydrogen peroxide, FRAP and inhibition of β-carotene degradation activity; diabetes was assayed on α-amylase and α-glucosidase activity	Antioxidant and antidiabetic potential	[63,64]
Total phenolics and flavonoids	In vitro	55–119 μg/mL	-	Assay of DPPH or ORAC, anti-inflammatory activities on LOX, COX-1, COX-2 pathways	Antioxidant and anti-inflammatory activities	[65]
Total phenols, flavonoids and tannins	In vitro	-	-	Assay of DPPH	Antioxidant potential	[66]
Total phenolics and flavonoids	In vitro	25 and 40 μM	Arsenic (10, 25, and 40 μM	Assay of transcriptional upregulation of serine acetyltransferase, *O*-acetyl serine (thiol)-lyase, γ-glutamylcysteine synthetase and phytochelatin synthase genes; assay of SOD, ascorbate peroxidase, dehydroascorbate reductase, GR and GST	Antioxidant potential	[67]
Hydroxybenzoic compounds, protocatechuic, vanillic acid, aldehyde *p*-hydroxybenzoic, trans-ferulic acid and trans-*p*-coumaric acid	In vitro	0.02 and 0.1% of lentil seed extracts	-	Assay of hydroxyl radical scavenging activity	Antioxidant potential	[68]
Kaempferol glucoside	In vitro	0.00625–5 mg/mL	-	Assay of DPPH, TEAC, FRAP and ORAC	Antioxidant potential	[33,69]
Total phenolics and flavonoids	In vitro	0.00625–5 mg/mL	-	Assay of DPPH	Antioxidant potential	[70,71]
Flavonol glycosides and free flavanols	In vitro	100 mg	-	Assay of PRTC, TEAC, ABTS, total phenolics, tocopherols (α-T, β-T, γ-T, δ-T), GSH and L-ascorbic acid	Antioxidant potential	[72,73]
Total phenolics and flavonoids	In vitro	20–100 μg/mL	-	Assay of COX-2 producing PGE (2) inhibitory assay	Anti-inflammatory activity	[74]

MTT: 3-(4,5-dimethylthiazol-2-yl)-2,5-diphenyltetrazolium bromide; ROS: reactive oxygen species; COX: cyclooxygenase; IL: interleukin; TNF: tumor necrosis factor; TEAC: trolox equivalent antioxidant capacity; LOX: lysyl oxidase; SOD: superoxide dismutase; GR: glutathione reductase; GST: glutathione s-transferase.

**Table 6:** Summary of in vitro, in vivo, clinical and intervention/observational studies on the anti-obesity and cardioprotective potentials of polyphenol-rich lentils.

Polyphenol-Rich Lentils	Model	Dose and Route of Administration	Negative Control	Investigation	Results	Reference
Flavonoids	Human colonic carcinoma cell line (Caco-2)	1.5, 3, 4.5, 6, 7.5 and 10 mg/mL	-	Assay of LDH, caspase-3, total DNA fragmentation, morphological changes related to apoptosis	Chemo-preventive agents	[75]
Free flavanols	Human with hyperhomocysteinemia and coronary artery disease	500 μg folate and 10 g lentils and other pulses and foods/p.o.	-	Assay of plasma total homocysteine	Cardioprotective activity	[76]
Phenolic acids	In vitro	20–100 μg/mL	-	Assay of platelet aggregation activity	Cardioprotective activity	[74]
Total phenolics	Male Wistar rats	200 and 400 mg/kg/p.o.	Doxorubicin (15 mg/kg bw/i.p.	Assay of BUN, serum creatinine, serum total protein, urinary total protein, and urinary creatinine, SOD, CAT, LPO and GSH in kidney	Nephroprotective potential	[77]
Phenolic compounds	Male albino rats	100, 200, 400 mg/kg/p.o.	-	Assay of blood picture (RBC, WBC and Hb), lipid fraction (total lipid, TC, TG, HDL, LDL and VLDL), liver function (AST, ALT and ALP, bilirubin) and kidney function (uric acid, urea and creatinine), total protein and its fractions (albumin and globulin), lipid peroxidation and antioxidative enzyme activity (SOD, CAT)	Hypolipidemic and antihypercholesterolemic activity	[78]
Total phenolics and flavonoids	Male Sprague-Dawley rats	Ten isocaloric and isonitrogenous diets were prepared; 5 of them were cholesterol-free and differed in the content of lentil powder (%): lentil-free (0), raw dehulled (60.5), raw whole (66.6), cooked dehulled (62.5) and cooked whole (65.6); while in the other 5, cholesterol (1%)	High cholesterol feed	Assay of TC, LDL-C, HDL-C, TG, AIP, CRR and atherogenic coefficient	Cardioprotective activity	[79]
Total phenolics	Male Wistar rats	200 g/kg/p.o. for 28 days	-	Assay of hepatic lipase and lipoprotein lipase in epididymal fat, gastrocnemius and heart	Cardioprotective and hypolipoproteinemia activity	[80]
Flavonoids	Sprague-Dawley female rats	100, 200, 400 mg/kg/p.o.	Triton WR-1339 (250 mg/kg/i.v.)	Assay of TC, TG, HDL, LDL and VLDL	Antihyperlipidemic activity	[81]
Total phenolics	Human	-	-	Cross-cultural and intervention studies	Cardioprotective activity	[82]
Phenolic acids	Human	13% p.o.	-	Assay of LDL	Hypolipidemic activity	[83]
Total phenolics	Human	120–130 g cooked lentil/day for 30–56 days/p.o.	-	Assay of TC, LDL, TG	Hypolipidemic activity	[84]
Phenolic acids	Human with hyperlipidemic patients	140 g/oral for 4 months’ time	-	Assay of serum TC and TG	Hypolipidemic activity	[85]

LDH: lactate dehydrogenase; BUN: blood urea nitrogen; CAT: catalase; LPO: lipid peroxidation; WBC: white blood cells; Hb: hemoglobin; HDL: high density lipoprotein; LDL: low density lipoprotein; VLDL: very low density lipoprotein; AST: aspartate transaminase; ALT: alanine transaminase; ALP: alkaline phosphatase; AIP: atherogenic index of plasma; CRR: cardiac risk ratio; i.v.: intravenous.

**Table 7:** Summary of the in vitro antimicrobial potentials of polyphenol-rich lentils.

Polyphenol-Rich Lentils	Model	Dose and Route of Administration	Negative Control	Investigation	Results	Reference
Flavonoids and lectins	*Staphylococcus aureus*, *Bacillus subtilis*, *Escherichia coli* and *Pseudomonas aeruginosa*	0.1–1 mL	-	Assay of agar well diffusion method	Antibacterial activity	[92]
Flavonoids	*Xanthomonas axonopodis* pv. *phaseoli*	250 mg/mL	-	Assay of disc diffusion method	Antibacterial activity	[90]
Ellagic acid, lupeol and leucodelphinidin	*Bacillus cereus*, *S. aureus*, *P. aeruginosa* and *E. coli*	250 mg/mL	-	Assay of disc diffusion method	Antibacterial activity	[93]
Flavonoids and proteins	*Aspergillus niger*	-	-	47-residue, plant defensin was purified by ammonium sulfate precipitation, gel filtration, chromatography and RP-HPLC; complete amino acid sequence, RT-PCR, cloning and cDNA sequence were performed	Antifungal activity	[21,91]
Flavonoids and proteins	*Fusarium oxysporum*	36 µM	-	Mycelial growth in *Mycosphaerella arachidicola*	Antifungal activity	[94]
Flavonoids, lentil lectin and the diterpene ester	Human peripheral blood mononuclear leucocytes. murine splenocytes and white Swiss inbred C67B1/6 mice	600 µg/mL	Concanavalin A	Assay of interferon-γ production	Antiviral activity	[95,96]

pv.: pathovar; RP-HPLC: reverse phase high performance liquid chromatography.

**Table 8:** Summary of in vitro, in vivo and clinical studies on the anticancer and chemopreventive effects of polyphenol-rich lentils.

Polyphenol-Rich Lentils	Model	Dose and Route of Administration	Negative Control	Investigation	Results	Reference
Flavonoids, lentil lectins	Human colon adenocarcinoma HT29 and colonic fibroblast CCD-18Co cells	19 µM	-	cDNA, encoding a Bowman–Birk protease inhibitor, assessed with an array of molecular masses	Antiproliferative properties in colon cancer	[97]
Flavonoids, lentil lectins	Human colon carcinoma cell line CACO-2	1.5, 3, 4.5, 6, 7.5 and 10 mg/mL	-	Production of IL-1, IL-6, IL-8 and MCP-1 were measured by ELISA and RT-PCR	Anticancer activity	[105]
Flavonoids, lentil lectins	Nasopharyngeal carcinoma CNE1 and CNE2 cell lines	1–5 mg/mL	-	Assay of MTT, flow cytometry and Western blotting	Anticancer activity	[106]
Total phenolics and flavonoids	In vitro	100 µL	2,2′-Azo*bis* (2-amidino propane hydrochloride	Assay of DPPH, radical scavenging assay, the hydroxyl radical- and the peroxyl radical-induced DNA strand scission assays	Potent chemopreventive agents	[100]
Cooked Lentil seeds with iron	Sprague-Dawley rats	35 mg/kg/p.o.	Iron-free diet (anemic group)	Assay of body weight, feed intake, Hb, hematocrit, MCV, MCH, MCHC, RBC, WBC and serum iron, platelet count and TIBC	Protective effect on iron deficiency anemia	[13]
Kaempferol, quercetin and myricetin	Human	1 cup cooked lentil/day/p.o.	-	Validated food frequency questionnaires in 1991 and 1995 from 90,630 women in the Nurses’ Health Study II	Protective against breast cancer	[98]
Flavonols	Human	1 cup cooked lentil/day/p.o.	-	Validated food frequency questionnaires	Protective against breast cancer	[107]
Total phenolics and flavonoids	Human	1 cup cooked lentil/day/p.o.	-	Validated food frequency questionnaires in 1976 and 1982 from 78,000 men	Protective against prostate cancer	[108]
Total phenolics and flavonoids	Human	1 cup cooked lentil/day/p.o.	-	Validated food frequency questionnaires in 617 incident cases of prostate cancer	Protects against prostate cancer	[109]
Isoflavones-genistein	Human	1 cup cooked lentil/day/p.o.	-	A validated food frequency questionnaires incident cases of prostate cancer	Protects against prostate cancer	[99]
Flavonols, flavones and flavonoid	Human	1 cup cooked lentil/day/p.o.	-	A validated food frequency questionnaires	Protects against prostate cancer	[110]
Flavonoids, lentil seed lectins	Human	-	-	Assay by using a flow cytometer	Screening for colorectal cancer	[111]
Flavonoids, lentil seed lectins	Patients with benign thyroid disease and thyroid carcinomas	-	-	Assay of *Lens culinaris* agglutinin reactive thyroglobulin ratios in sera and wash fluids	Useful for distinguishing between thyroid carcinoma and benign thyroid tumor	[112]
Flavonoids, lentil seed lectins	Patients with benign thyroid disease and thyroid carcinomas	-	-	Assay of *Lens culinaris* agglutinin reactive thyroglobulin ratios in sera and wash fluids	Useful prognostic marker for thyroid cancer	[113]

MCP: monocyte chemotactic protein; MCV: mean corpuscular value; MCH: mean corpuscular hemoglobin; MCHC: mean corpuscular hemoglobin concentration; TIBC: total iron binding capacity.

## Abbreviations

ABTS	2,2′-azino-bis(3-ethyl-benzothiazoline-6-sulphonic acid)
AFP	α-fetoprotein
AIP	atherogenic index of plasma
ALP	alkaline phosphatase
ALT	alanine transaminase
AST	aspartate transaminase
bw	body weight
BP	blood pressure
BUN	blood urea nitrogen
CAT	catalase
cDNA	complementary deoxyribonucleic acid
COX-1, 2	cyclooxygenase 1, 2
CVD	cardiovascular diseases
CRR	cardiac risk ratio
DNA	deoxyribonucleic acid
DPPH	2,2-diphenyl-1-picrylhydrazyl
ELISA	enzyme-linked immunosorbent assay
FBS	fasting blood sugar
FRAP	ferric reducing antioxidant power assay
GR	glutathione reductase
GSH	reduced glutathione
GST	glutathione-s-transferase
HbA1C	glycated hemoglobin
Hb	hemoglobin
HDL	high density lipoprotein
HPLC	high performance liquid chromatography
i.p.	intraperitoneal
i.v.	intravenous
IL	interleukin
kg	kilogram
LDH	lactate dehydrogenase
LDL	low density lipoprotein
LOX	lysyl oxidase
LPO	lipid peroxidation
MCH	mean corpuscular hemoglobin
MCHC	mean corpuscular hemoglobin concentration
MCP-1	monocyte chemotactic protein 1
MCV	mean corpuscular value
MTT	3-(4,5-dimethylthiazol-2-yl)-2,5-diphenyltetrazolium bromide
OGTT	oral glucose tolerance test
ORAC	oxygen radical absorbance capacity
p.o.	per oral
PGE (2)-PRTC	peroxyl radical-trapping capacity
RBC	erythrocyte
ROS	reactive oxygen species
RP	reducing power
RT-PCR	reverse transcriptase polymerase chain reaction
SOD	super oxide dismutase
STZ	streptozotocin
TC	total cholesterol
TEAC	trolox equivalent antioxidant capacity
TG	triglycerides
TIBC	total iron binding capacity
TNF-α	tumor necrosis factor alpha
VLDL	very low density lipoprotein
WBC	leucocyte
